# Integrated Seamless Non-Noble Plasmonic Ni-Upconversion Nanofilm for Stable and Enhanced Fluorescence Performance

**DOI:** 10.3390/ma18173995

**Published:** 2025-08-26

**Authors:** Hao Zeng, Longhui Han, Yang Li, Yaru Ni, Chunhua Lu

**Affiliations:** 1 State Key Laboratory of Materials-Oriented Chemical Engineering, College of Materials Science and Engineering, Nanjing Tech University, Nanjing 211816, China; zenghaoxbb@126.com (H.Z.); hlh1691553469@163.com (L.H.); chhlu@njtech.edu.cn (C.L.); 2 Jiangsu Collaborative Innovation Center for Advanced Inorganic Function Composites, Nanjing Tech University, Nanjing 211816, China; 3 Jiangsu National Synergetic Innovation Center for Advanced Materials (SICAM), Nanjing Tech University, Nanjing 211816, China

**Keywords:** upconversion, surface plasmon resonance, nanofilm, NaYF_4_, nanofabrication

## Abstract

Thickness-controlled, easily patterned upconversion (UC) nanofilms are essential for high-precision optoelectronic devices, but challenges such as imprecise thickness control and low fluorescence intensity hinder their application. High-performance lanthanide-doped sodium yttrium fluoride UC materials are typically available in powder form, making direct integration into advanced devices difficult. Although physical vapor deposition (PVD) enables precise film formation, it often produces poor crystalline structures and weak fluorescence. To overcome these limitations, integrating non-noble plasmonic Ni with surface plasmon resonance to enhance fluorescence intensity is a promising yet understudied strategy, likely due to Ni’s ultraviolet resonant wavelength and oxidation susceptibility. This study introduces an integrated Ni-UC nanofilm design, combining an ultrathin Ni layer with a NaYF_4_:Tm, Yb UC layer via PVD, followed by post-annealing. Annealing at 500 °C transforms the UC layer into a hexagonal-phase crystal structure while protecting the Ni layer from oxidation. The unannealed UC nanofilm showed no fluorescence, whereas the annealed UC nanofilm displayed clear peaks at 476, 648, and 699 nm. Notably, the integrated Ni-UC nanofilm exhibited fluorescence intensities 5.29, 4.43, and 4.29 times higher at these wavelengths, respectively. Additionally, the integrated design exhibited high transparency and stability, highlighting its protective benefits. These results underscore the potential of the integrated Ni-UC nanofilm for advanced optoelectronics and sensing technologies, offering enhanced fluorescence, micro-processing compatibility, and robust performance in a cost-effective, non-noble plasmonic system.

## 1. Introduction

Most photosensitive materials are sensitive to high-energy light such as ultraviolet and visible light, which only account for 45% of the solar spectrum [1]. Therefore, approximately 55% of the solar spectrum, consisting of near-infrared (NIR) light, remains underutilized. Upconversion (UC) nanomaterials can absorb low-energy NIR light and convert it into high-energy visible and ultraviolet light through a nonlinear anti-Stokes process [2]. These materials have garnered attention for their potential applications in bioimaging [3,4], therapy [5,6], photovoltaic cells [7,8], sensors [9], anti-counterfeiting security [10,11], and 3D displays [12,13]. Among them, sodium yttrium fluoride doped with lanthanide ions (NaYF_4_:Ln) is one of the most stable UC materials under NIR excitation [14], outperforming traditional organic UC materials, which suffer from low efficiency and instability under prolonged excitation [15].

Recently, forming UC materials into nanoscale films with controllable thickness and easy patterning has become increasingly important for integrating devices such as solar cells and 3D displays, especially for optical films requiring high precision [16,17]. However, most NaYF_4_:Ln materials are currently in powder form. Traditional methods for preparing UC films, such as nanoparticle self-assembly [18], electrodeposition [19,20], and sol–gel methods [21,22], face issues with imprecise thickness control, numerous defects, high surface roughness, and poor adhesion to substrates. These problems can be addressed by physical vapor deposition (PVD) technology, particularly in the field of high-precision optical films. However, PVD often results in poor crystalline structure formation, leading to low fluorescence intensity [23], which hinders the practical application of UC films in devices. Therefore, it is essential to investigate methods for optimizing the crystalline structure formation of UC nanofilms via PVD and to develop strategies that enhance fluorescence intensity while ensuring high compatibility in structural integration.

Over the past few decades, plasmonic metal nanostructures have provided promising solutions for improving light utilization through surface plasmon resonance (SPR) [24], which involves the collective oscillation of electrons in response to the oscillation of an incident light field. Plasmonic nanostructures enhance light utilization through multiple mechanisms: (1) localized electric field amplification for fluorescence upconversion, (2) modified local photonic density of states to accelerate radiative decay [25,26], (3) improved energy transfer between luminescent material and the metallic nanostructure [27], and (4) far-field light scattering. These effects collectively enable both direct and indirect optimization of light-harvesting efficiency. Recently, plasmonic metal nanostructures have been shown to effectively enhance the fluorescence intensity of UC materials [28]. Among various plasmonic metals, Au stands out for its superior SPR performance [29]. However, the high cost and limited availability of Au hinder its widespread practical application. In contrast, Ni, a non-precious metal, offers several advantages, including abundance, low cost, and high electron concentration, which could potentially lead to high SPR performance. However, Ni has been rarely studied for SPR-enhanced UC, if at all, likely due to its primary resonance wavelength in the ultraviolet range [30] and its propensity for oxidation, which compromises its stability.

**Figure 1:**
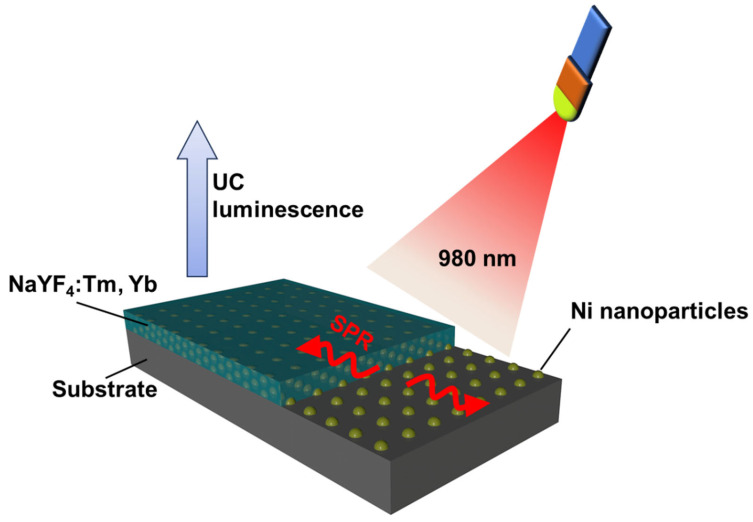
Schematic diagram illustrating the mechanism of the integrated Ni-UC nanofilm.

In this study, we present a seamless non-noble plasmonic integrated Ni-UC nanofilm design, as illustrated in Figure 1, comprising an ultrathin Ni layer and an upper NaYF_4_:Tm, Yb UC nanofilm layer, fabricated via vacuum thermal evaporation and integrated post-annealing processing. This design enables the transformation of the UC nanofilm layer into a crystalline form with enhanced fluorescence while simultaneously annealing the Ni nanofilm and protecting the Ni nanofilm from oxidation. We systematically investigate the effects of annealing temperatures on the crystal structure and fluorescence intensity of UC nanofilms, as well as the combined influence of annealing temperatures and Ni layer thickness on the fluorescence performance of the integrated Ni-UC nanofilms. Annealing at 500 °C results in the formation of a hexagonal-phase NaYF_4_ crystal in the UC layer. The 5 nm Ni layer exhibits significant SPR absorption at the 980 nm excitation wavelength and minimal absorbance in the UC emission region (400–800 nm), leading to markedly enhanced fluorescence in the integrated Ni-UC nanofilm. The as-deposited UC nanofilm fabricated via PVD without annealing exhibits negligible fluorescence intensity, whereas annealing induced distinct fluorescence peaks at 476, 648, and 699 nm. Remarkably, the integrated Ni-UC nanofilm demonstrates fluorescence intensities 5.29, 4.43, and 4.29 times those of the annealed individual UC nanofilm at the respective wavelengths. Moreover, the integrated Ni-UC nanofilm maintains at least 75% of its fluorescence intensity after 90 days, underscoring the stability of the design. These findings highlight the potential of the Ni-UC nanofilm for applications in optoelectronics and sensing technologies, where nanoscale, patternable UC films with stable and enhanced fluorescence are essential.

## 2. Experimental

**Figure 2:**
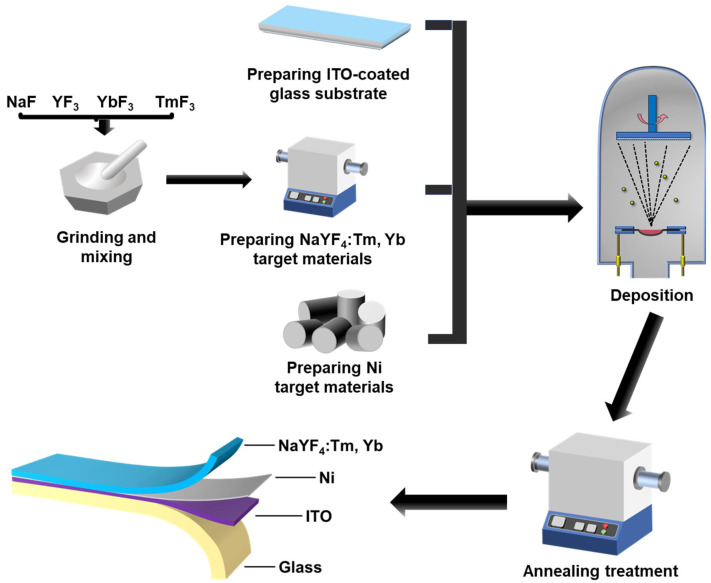
Schematic diagram showing the main processes for preparing NaYF_4_:Yb, Tm fluorescent films, and the integrated Ni-UC nanofilm system.

Figure 2 shows a schematic diagram of the main processes for preparing NaYF_4_:Tm, Yb fluorescent films, and the integrated Ni-UC nanofilm system.

### 2.1. Preparation of NaYF_4_:Tm, Yb Film Materials

The NaYF_4_:Tm, Yb fluorescent film coating target material was prepared using a high-temperature solid-phase method. The raw materials, NaF, YbF_3_, YF_3_, and TmF_3_ (Rhawn, Shanghai Easy Chemical Technology Co., Ltd., Shanghai, China), were mixed in a molar ratio of 1:0.835:0.15:0.015 and ground for 1 h. The mixed powder was placed into an Al_2_O_3_ crucible and then heated in a tube furnace under argon atmosphere at 900 °C for 6 h, followed by natural cooling to room temperature.

### 2.2. Preparation of Substrate Materials

Commercially available ITO glass (Shenzhen South Huacheng Technology Co., Ltd., Shenzhen, China) was used as the substrate. The Ni metal transition layer film on the ITO glass was prepared using a vacuum coating method, and the Ni target material (Fuzhou Infinium Photonics Co., Ltd., Fuzhou, China) consisted of cylindrical particles with both diameter and height of approximately 6 mm. The cut ITO glass was cleaned in deionized water and anhydrous ethanol, before being dried with a nitrogen gun. The ITO glass substrate and Ni target material in a tungsten crucible were placed in a vacuum coating machine (TEMD500, Beijing Technoway Technology Co., Ltd., Beijing, China). The Ni film was deposited using an electron beam, with no substrate baking temperature, a distance of about 400 mm between the substrate and target, and a deposition rate of 0.04 nm/s under a vacuum of 2 × 10^−3^ Pa.

### 2.3. Film Preparation and Post-Treatment Process

Using Tm, Yb-doped NaYF_4_ bulk as the target material, NaYF_4_:Tm, Yb films were prepared using vacuum thermal evaporation (the target material was prepared using the high-temperature solid-phase method). The NaYF_4_:Tm,Yb bulk was placed in a molybdenum boat, and the boat and prepared substrate were placed in the vacuum coating machine. The substrate baking temperature was 250 °C, and deposition was carried out under a vacuum of 5 × 10^−3^ Pa. The deposition rate was maintained at 0.3–0.4 nm/s, and the NaYF_4_:Tm,Yb film thickness was 350 nm. Film heat treatment was performed in a dual-zone atmosphere tube furnace (QTF-1200X, Hefei Kejing Materials Technology Co., Ltd., Hefei, China) under an argon atmosphere, with annealing occurring at 400 and 500 °C for 4 h.

### 2.4. Structural and Optical Characterization

The crystal structure of the fluorescent films was studied using an X-ray diffractometer (XRD, SmartLabTM, Rigaku, Japan). The surface morphology of the films was characterized using a scanning electron microscope (SEM, ZEISS-sigma300, Oberkohen, Germany). The surface elemental composition of the films was characterized using a photoelectron spectrometer (XPS, Thermo Fisher Scientific-K-Alpha, Waltham, MA, USA). Photoluminescence (PL) spectra were measured using a fluorescence spectrometer (FL3-221, HORIBA Jobin Yvon, Paris, France) under excitation from a 500 mW semiconductor infrared laser at 980 nm (λ_em_ = 980 nm). Fluorescence lifetime analysis was performed using a transient steady-state fluorescence spectrometer (FLS1000, Edinburgh Instruments, Edinburgh, UK). The basic optical properties of the films were characterized using an ultraviolet-visible spectrophotometer (Agilent Cary 5000, Agilent, Beijing, China).

### 2.5. FDTD (Finite-Difference Time-Domain) Simulation

Numerical simulations of the electric field profile were performed using commercial software (Lumerical FDTD Solutions 2018a) based on the FDTD technique. In the excitation enhancement simulation, the dielectric function of Ni was based on experimental data measured by Palik, and the dielectric data for NaYF_4_:Tm,Yb were obtained from our measurements of pure fluorescent films. The structure was set with periodic boundary conditions in the x and y directions and perfectly matched layer boundary conditions in the z-axis direction. A 980 nm plane wave source was used as the incident light in the simulation.

## 3. Results and Discussion

**Figure 3:**
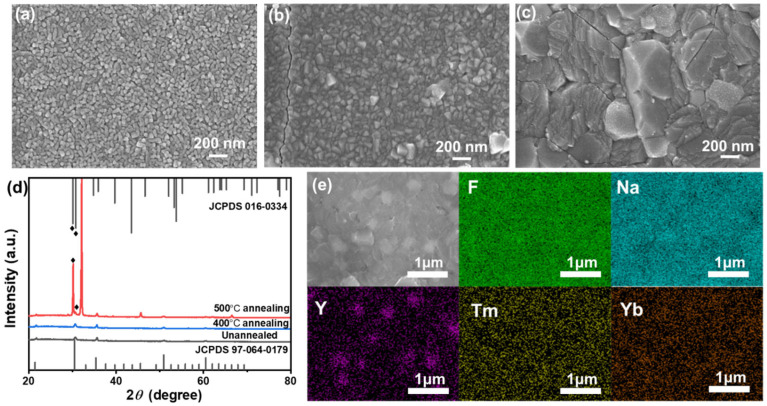
SEM images of NaYF_4_:Tm,Yb nanofilms (**a**) unannealed, (**b**) annealed at 400 °C, and (**c**) annealed at 500 °C. (**d**) XRD patterns of NaYF_4_:Tm,Yb nanofilms under different annealing temperatures. EDS image of (**e**) the surface of the NaYF_4_:Tm, Yb UC nanofilm annealed at 500 °C.

A 350 nm thick NaYF_4_:Tm, Yb single-layer nanofilm was prepared using the PVD method and subjected to different annealing conditions: no annealing, 400 °C annealing, and 500 °C annealing. Surface SEM analysis (Figure 3a–c and Figure S1) revealed distinct morphological changes in the UC nanofilm under varying annealing treatments. The as-deposited UC nanofilm exhibits a surface composed of nanorods with average feature size of 39.6 nm. After 4 h of annealing at 400 °C, the nanorods begin to interconnect, and become well-defined particles with average feature size of 58.0 nm. Further annealing at 500 °C for 4 h results in a sintered surface morphology, characterized by the probable formation of crystalline particles with average feature size of 326.3 nm. The average particle size is obtained from the statistics in Figure S2. The observed morphological evolution is likely attributed to the increased thermal kinetic energy during annealing, which facilitates grain boundary expansion and particle growth.

To verify the changes in the crystallization of the UC films, XRD analysis was conducted on samples under different annealing conditions. As depicted in Figure 3d, the NaYF_4_:Tm, Yb nanofilms remain amorphous in their as-prepared state and even at lower annealing temperatures (400 °C). The crystalline peaks observed in the spectrum correspond to the standard reference card JCPDS 97-064-0179 [20,31], indicating that these minor crystalline peaks originate from the ITO layer of the substrate. However, when the annealing temperature is increased to 500 °C, new XRD peaks appear at 2θ = 30.5°, corresponding to the standard hexagonal NaYF_4_ (JCPSD 16-0334) [20,31], confirming the crystallization of NaYF_4_:Tm, Yb annealed at 500 °C. The XRD diffraction peak observed at 2θ = 32.1° corresponds to the standard cubic phase NaYF_4_ (JCPSD 06-0342) [20,31], indicating the coexistence of cubic and hexagonal phases in NaYF4 after 500 °C annealing treatment. Additionally, as shown in Figure 3e, the main elements (Na, Y, F, Yb, Tm) of the UC material are uniformly distributed on the film surface.

**Figure 4:**
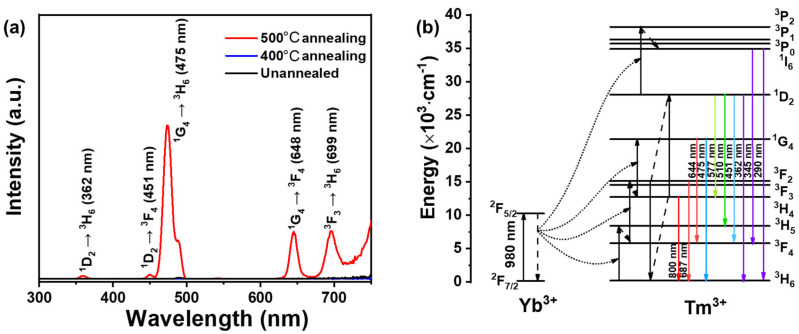
(**a**) Fluorescence emission spectrum of the UC nanofilm (λ_em_ = 980 nm). (**b**) UC emission mechanism in the Yb^3+^ and Tm^3+^ co-doped system.

The UC properties of the abovementioned NaYF_4_:Tm, Yb nanofilms were also investigated. As shown in Figure 4a, only the UC nanofilm annealed at 500 °C shows significant fluorescence peaks. In Figure 4b, the fluorescence peaks correspond to the energy level transitions of Tm [32]: ^1^D_2_→^3^H_6_ (362 nm), ^1^D_2_→^3^F_4_ (451 nm), ^1^G_4_→^3^H_6_ (475 nm), ^1^G_4_→^3^F_4_ (648 nm), and ^3^F_3_→^3^H_6_ (699 nm). In the upconversion luminescence system of Yb^3+^ and Tm^3+^, Yb^3+^ acts as a sensitizer, possessing a large absorption cross-section and a long excited-state lifetime, which enables efficient energy transfer. It is primarily responsible for absorbing 980 nm NIR light and transferring the energy to Tm^3+^. Tm^3+^ serves as an activator, receiving the energy transferred from Yb^3+^ and achieving upconversion luminescence through a multi-photon process. Due to the rich energy levels of Tm^3+^, it can emit light of various colors, which also explains the luminescence mechanism of UC nanofilms.

Compared to crystalline materials, amorphous materials have more defects, which can interfere with energy transfer between ions in the film, increasing the probability of non-radiative transitions in UC materials [33,34,35]. Crystallized UC materials have fewer defects, improving UC capability. According to the XRD results, the crystallinity increased in the order of no annealing, 400 °C annealing, and 500 °C annealing, consistent with the fluorescence intensity trends. After annealing at 500 °C, the nanofilm showed good crystallinity and strong fluorescence intensity, while the films not subjected to annealing and those annealed at 400 °C showed weak crystallinity and almost no fluorescence.

**Figure 5:**
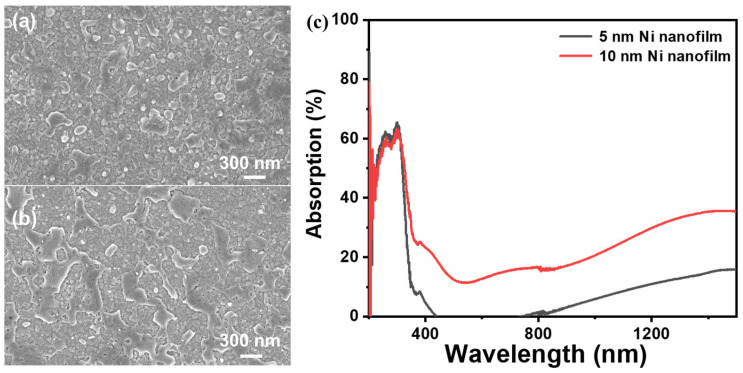
SEM images of (**a**) 5 nm Ni film and (**b**) 10 nm Ni film. (**c**) Absorption spectra of Ni films with different thicknesses.

**Figure 6:**
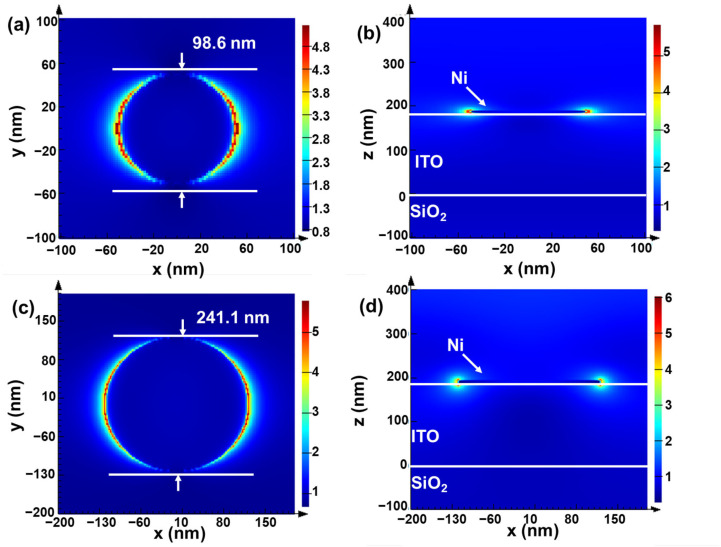
FDTD-simulated electric field distribution: (**a**,**b**) electric field distribution of the 5 nm Ni film; (**c**,**d**) electric field distribution of the 10 nm Ni film.

Additionally, we attempted to use Ni as a non-precious metal plasmonic material to enhance UC luminance. To study the plasmonic effects of Ni, we prepared 5 and 10 nm Ni nanofilms and annealed them at 500 °C for 4 h. It can be seen that after high-temperature annealing, the 5 nm Ni film formed small nano-islands arranged in a non-continuous, island-like growth pattern, as shown in Figure 5a and Figure S3a, with particles about 98.6 nm in diameter and spaced about 118.3 nm apart (Figure S4). The 10 nm Ni film shows a continuous pattern, with particles about 241.1 nm in diameter and long strips about 195.0 nm wide, as shown in Figure 5b and Figure S3b. The average particle size is obtained from the statistics in Figure S4. The calculation method of the absorptivity of Ni nanofilms is shown from Figure S5. As shown in Figure 5c, the absorption spectrum of the Ni nanofilm demonstrates strong absorption characteristics in the ultraviolet range of 200–380 nm. At the 300 nm wavelength, the Ni nanofilms with thicknesses of 5 and 10 nm reach their highest absorption rates of 65% and 63%, respectively. In the range of 400–800 nm, which primarily covers the upconversion emission band, the absorption is low. The average absorption rate of the 10 nm Ni nanofilm in this range is 15%, while the absorption of the 5 nm Ni nanofilm is almost negligible. Above 800 nm, including the 980 nm upconversion excitation band, the absorption is high. At the 980 nm excitation wavelength, the absorption of the 10 nm Ni nanofilm reaches 21%, and the absorption of the 5 nm Ni nanofilm also increases to 6%, which is beneficial for enhancing the UC luminance. The SPR effect of the Ni nanofilm is demonstrated via the FDTD-simulated electric field distribution, modeling the annealed Ni film particles as disks. The structure of the simulated Ni nanofilms is shown in Figure S6. As illustrated in Figure 6a–d, the simulation results demonstrate significant plasmonic enhancement at the edges of the nano-disks. The electric field around the Ni nano-disks is amplified by approximately six times, localized within 20 nm of the nano-disks. Simulations of this structure under 980 nm parallel light irradiation reveal that the 5 and 10 nm Ni layers exhibit localized surface plasmon resonance (LSPR) electric field enhancement, confirming the plasmonic effects of the Ni nanofilms. In the future, we will further explore the correlation between the diameter of Ni nano-disks and the LSPR peaks. We also added a 350 nm thick NaYF_4_:Tm, Yb layer on top of the 5 nm Ni nanofilm. The refractive index data were obtained via measurement of the single-layer NaYF_4_:Tm, Yb film (Figure S7). As shown in Figure S8, there is an electric field enhancement of 980 nm in the region of UC nanofilm.

**Figure 7:**
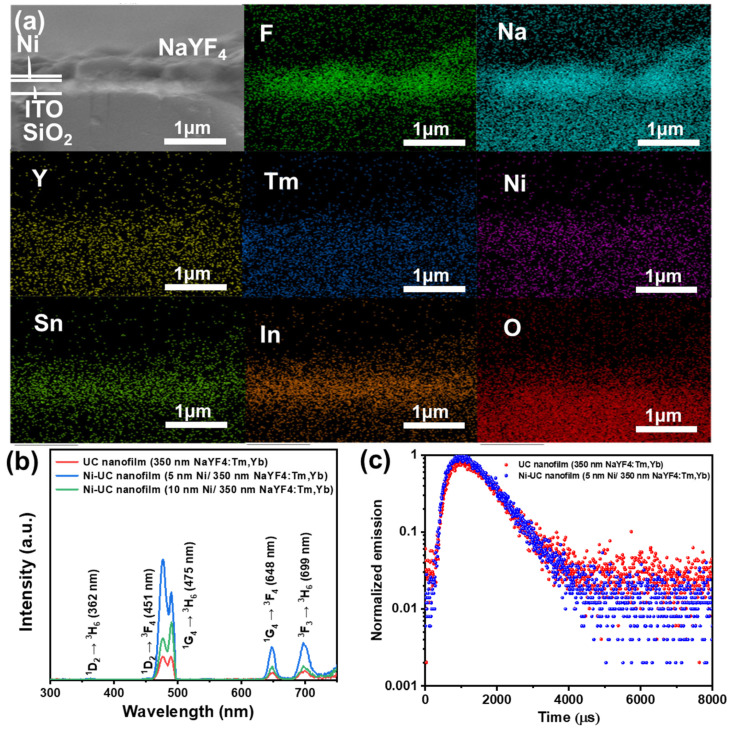
(**a**) Cross-sectional EDS mapping of the integrated Ni-UC nanofilm (5 nm Ni/350 nm NaYF_4_:Tm, Yb). (**b**) Fluorescence emission spectra of UC films composited with Ni films of different thicknesses (λ_em_ = 980 nm). (**c**) Fluorescence lifetime curves of the UC film and the integrated Ni-UC nanofilm (5 nm Ni/350 nm NaYF_4_:Tm, Yb).

To evaluate the effectiveness of Ni nanofilms with SPR in enhancing upconversion, we fabricated 350 nm UC nanofilms integrated with 5 nm and 10 nm Ni layers. As shown in Figure 7a, the film layers are uniform and clear, indicating successful preparation of the composite films. As shown in Figure 7b, the 5 nm Ni film enhances the fluorescence peaks at 476, 648, and 699 nm by 5.29, 4.43, and 4.29 times, respectively, while the 10 nm Ni film enhances the peaks by 1.82, 1.69, and 1.58 times, respectively. The FDTD simulation shows that the 10 nm Ni film has slightly stronger electric field enhancement than the 5 nm Ni film. The observed difference in fluorescence intensity can be attributed to two primary factors. Firstly, the 10 nm Ni film exhibits strong absorption within the three main emission bands, leading to partial fluorescence quenching. Secondly, the enhancement of the electric field by the LSPR effect of metal nano-islands decays exponentially with the distance from the surface of the metal nano-islands. From the simulated electric field diagrams (Figure 6a–d), it can be observed that the enhancement effect of LSPR is predominantly localized within 20 nm from the surface of the metal nano-islands [36]. Within the same area, the 5 nm Ni nanofilm contains a significantly higher density of nano-islands compared to the 10 nm Ni nanofilm, as evidenced by the surface SEM images of the Ni nanofilms (Figure 5a,b). Consequently, the specific surface area of the 5 nm Ni nanofilm is much larger than that of the 10 nm Ni nanofilm, enhancing the ability of Ni with SPR to interact with UC materials across a broader region. This explains why the fluorescence intensity of the integrated Ni-UC nanofilm (5 nm Ni/350 nm NaYF_4_:Tm, Yb) is stronger than that of the integrated Ni-UC nanofilm (10 nm Ni/350 nm NaYF_4_:Tm, Yb). To explore the mechanism of Ni nanofilms in enhancing fluorescent films, we tested the fluorescence lifetime of single-layer fluorescent films and 5 nm Ni film-composited fluorescent films. As shown in Figure 7c, the average fluorescence lifetime (τ_ave_) of the 5 nm Ni film-composited fluorescent film is 835.8 μs, while that of the single-layer fluorescent film is 916.7 μs. Combining the LSPR effects of Ni particles, we speculate that higher-intensity excitation light increases the population of excited-state molecules, increasing the probability of collisions between molecules and, thus, the probability of non-radiative transitions, leading to shorter fluorescence lifetimes [37,38].

**Figure 8:**
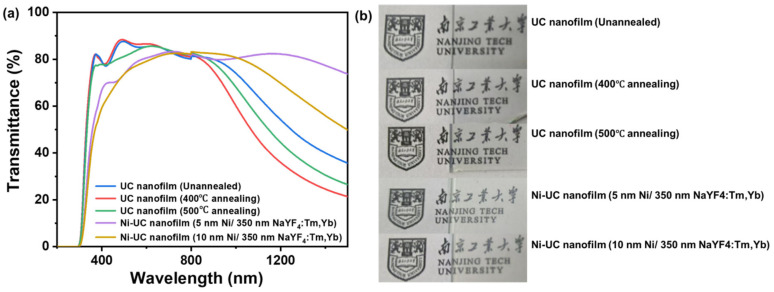
(**a**) Transmittance spectra and (**b**) photographs of different UC nanofilms and integrated Ni-UC nanofilms (the photographs’ backgrounds show the logo of Nanjing Tech University, with a width of 1 cm).

**Figure 9:**
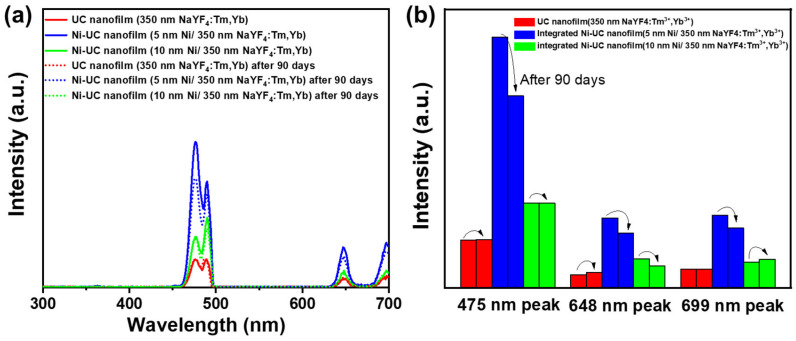
(**a**) Fluorescence emission spectra of the UC film and the integrated Ni-UC nanofilms after 90 days of storage (λ_em_ = 980 nm); (**b**) comparative intensities of the three dominant fluorescence emission peaks (475 nm, 648 nm, 699 nm) for the UC film and integrated Ni-UC nanofilms after 90 days of storage.

For practical application of nanofilms, transparency and stability are also important characteristics. As shown in Figure 8, in the visible light region of 370–750 nm, which is also the primary region for UC luminescence, the average transmittance of the samples shown in the figure is 84%, 84%, 82%, 77%, and 76%, respectively, indicating relatively high transparency. This demonstrates that the samples exhibit good transparency in the visible light region, ensuring the transmission of UC luminescence. In the NIR region, the transmittance of the nanofilms decreases to varying degrees. The fastest decline was observed in, with a transmittance of 63% at 980 nm, followed by the UC nanofilm (500 °C annealing), with a transmittance of 70% at 980 nm. The UC nanofilm (unannealed) ranks third, with a transmittance of 75% at 980 nm, while the integrated Ni-UC nanofilm (10 nm Ni/350 nm NaYF_4_:Tm, Yb) has a transmittance of 82% at 980 nm. Finally, the integrated Ni-UC nanofilm (5 nm Ni/350 nm NaYF_4_:Tm, Yb) exhibits slight transmittance attenuation at 980 nm, with transmittance settling at 80%. Additionally, as shown in Figure 9, after 90 days of storage (temperature: 12–13 °C; relative humidity: ~78%), we observed that the three main fluorescence peaks of the UC nanofilm and integrated Ni-UC nanofilm (10 nm Ni/350 nm NaYF_4_:Tm, Yb) retained more than 99% of their intensity. For the integrated Ni-UC nanofilm (5 nm Ni/350 nm NaYF_4_:Tm, Yb), the fluorescence peak at 475 nm maintains 75% of its intensity, the peak at 648 nm retains 77% of its intensity, and the peak at 699 nm retains 80% of its intensity. Repeated fluorescence emission measurements showed that the films retained at least 75% of their fluorescence intensity, indicating good stability over time. To evaluate the fluorescence properties of the UC nanofilms, we conducted imaging to capture their actual fluorescence performance. As illustrated in Figure S9, the UC nanofilm without annealing and that annealed at 400 °C exhibit negligible fluorescence. In contrast, the nanofilm annealed at 500 °C and the UC nanofilms integrated with 5 nm and 10 nm Ni layers demonstrate bright and distinct fluorescence.

## 4. Conclusions

In summary, we propose a seamless non-noble plasmonic integrated Ni-UC nanofilm design, fabricated via vacuum thermal evaporation and post-annealing processing, to achieve stable, high-performance, thickness-controlled, and easily patterned UC nanofilms. The effects of annealing temperature on NaYF_4_:Tm, Yb nanofilms prepared by PVD were systematically investigated, revealing an optimal annealing temperature of 500 °C, which facilitates the transformation of amorphous NaYF_4_ into its hexagonal phase. During annealing, the Ni nanofilm is simultaneously converted into nano-islands, which are protected by the overlying UC nanolayer. This configuration not only provides an SPR effect, enhancing upconversion luminescence performance by 5.29 times compared to the single-layer UC nanofilm, but it also ensures high stability and visible transparency. Theoretical simulations using the FDTD method further corroborate the experimental observations regarding the SPR effect. These findings validate the feasibility of employing PVD to fabricate thickness-controllable UC film devices and highlight the potential of Ni as a cost-effective non-precious plasmonic material. The integrated Ni-UC nanofilm enables precise thickness control and stable, enhanced fluorescence, opening new possibilities for applications in bioimaging, anti-counterfeiting, solar cells, and optoelectronic sensors, while its tunable properties provide critical insights for designing advanced optoelectronic and sensing devices.

## Data Availability

The original contributions presented in this study are included in the article/Supplementary Material. Further inquiries can be directed to the corresponding author(s).

## Supplementary Materials

The following supporting information can be downloaded at: https://www.mdpi.com/article/10.3390/ma18173995/s1, Figure S1: Surface SEM images of UC nanofilms at different magnifications (a,b) unannealed, (c) annealed at 400 °C and (d,e) annealed at 500 °C.; Figure S2: Surface particle size distribution map of UC nanofilms (a) unannealed, (b) annealed at 400 °C and (c) annealed at 500 °C.; Figure S3: Surface SEM images of Ni nanofilms at different magnifications (a) 5 nm Ni film and (b) 10 nm Ni film.; Figure S4: Surface particle size distribution map of Ni nanofilms (a) 5 nm Ni film and (b) 10 nm Ni film.; Figure S5: Measurement of optical properties of Ni nanofilms: (a) transmittance, (b) reflectance.; Figure S6: Schematic diagram of Ni nanofilms structure used in FDTD electric field simulation: (a) 5 nm Ni nanofilm, (b) 10 nm Ni nanofilm.; Figure S7: Measurement of optical constants of NaYF4:Tm, Yb nanofilm: (a) n, (b) k.; Figure S8: FDTD electric field simulation of the integrated Ni-UC nanofilm (5 nm Ni/350 nm NaYF4: Tm, Yb).; Figure S9: Photographs of fluorescence emission of different UC nanofilms and integrated Ni-UC nanofilms (we used a commercially available 100 mW laser pen emitting 980 nm laser to irradiate the UC nanofilms, filtered out the infrared light with a visible light filter, and photographed the UC nanofilms): (a) UC nanofilm (Unannealed), (b) UC nanofilm (400 °C annealing), (c) UC nanofilm (500 °C annealing), (d) Ni-UC nanofilm (5 nm Ni/350 nm NaYF_4_:Tm, Yb), (e) Ni-UC nanofilm (10 nm Ni/350 nm NaYF_4_:Tm, Yb).
