# Supplementary material for: Integrated Seamless Non-Noble Plasmonic Ni-Upconversion Nanofilm for Stable and Enhanced Fluorescence Performance
Source: Materials (Basel). 2025 Aug 26;18(17):3995. doi: 10.3390/ma18173995 (PMC12429482; doi:10.3390/ma18173995)
Supplement: Supplementary file 1 [file materials-18-03995-s001.zip › materials-3797400-supplementary.pdf]

materials

Article

Integrated Seamless Non-Noble Plasmonic Ni-Upconversion Nanofilm for Stable and Enhanced Fluorescence Performance

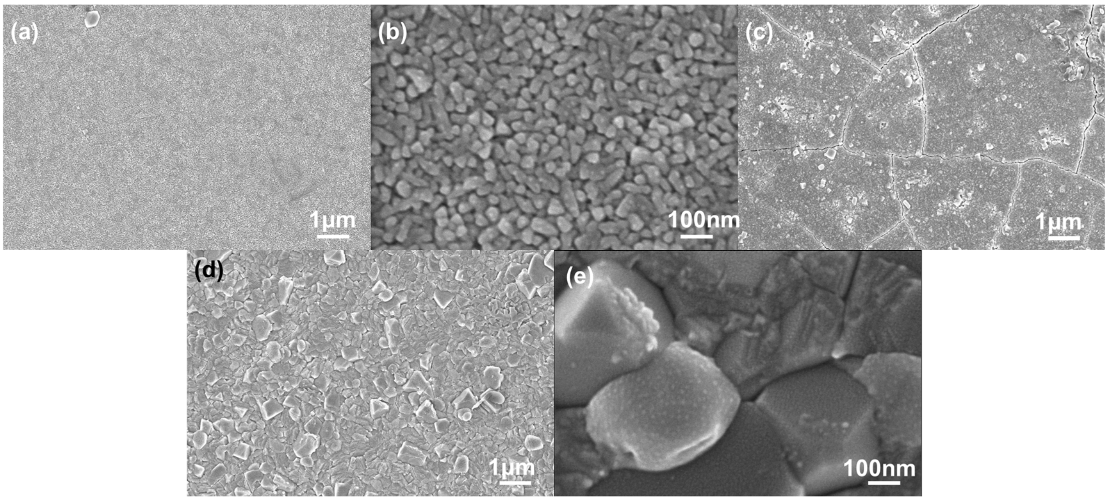

Figure S1. Surface SEM images of UC nanofilms at different magnifications (a-b) unannealed, (c) annealed at 400℃ and (d-e) annealed at 500℃.

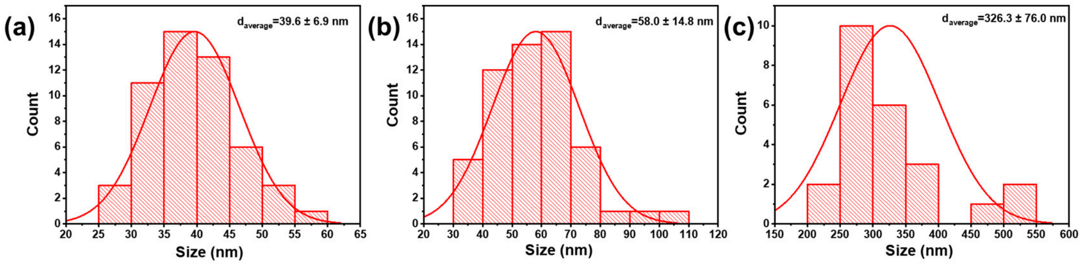

Figure S2. Surface particle size distribution map of UC nanofilms (a) unannealed, (b) annealed at 400℃ and (c) annealed at 500℃.

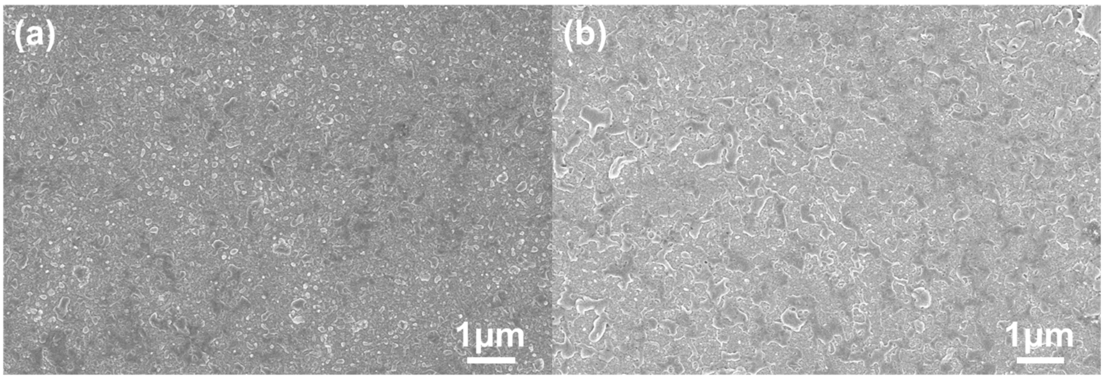

Figure S3. Surface SEM images of Ni nanofilms at different magnifications (a) 5 nm Ni film and (b) 10 nm Ni film.

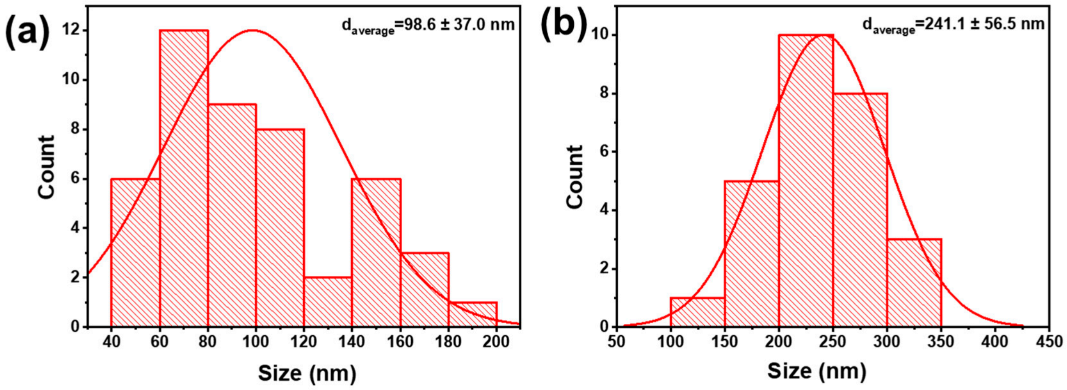

Figure S4. Surface particle size distribution map of Ni nanofilms (a) 5 nm Ni film and (b) 10 nm Ni film.

The absorption of Ni nanofilm is obtained from the following formula:
A=1-T-R

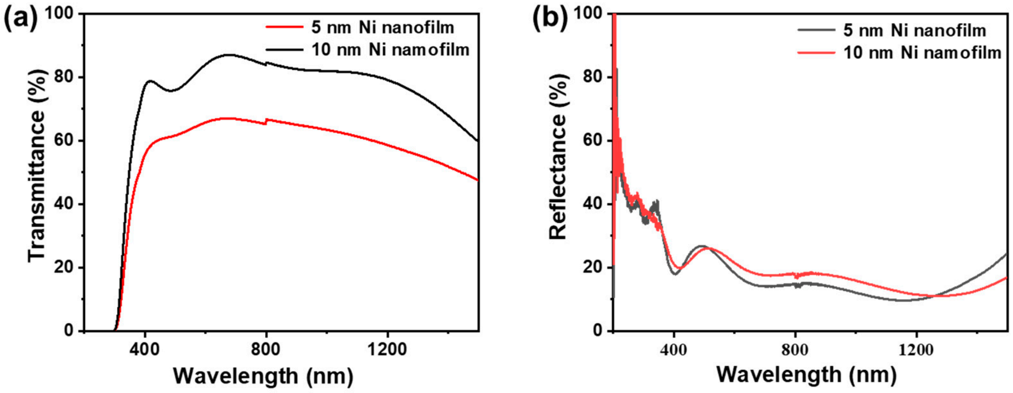

Figure S5. Measurement of optical properties of Ni nanofilms: (a) transmittance, (b) reflectance.

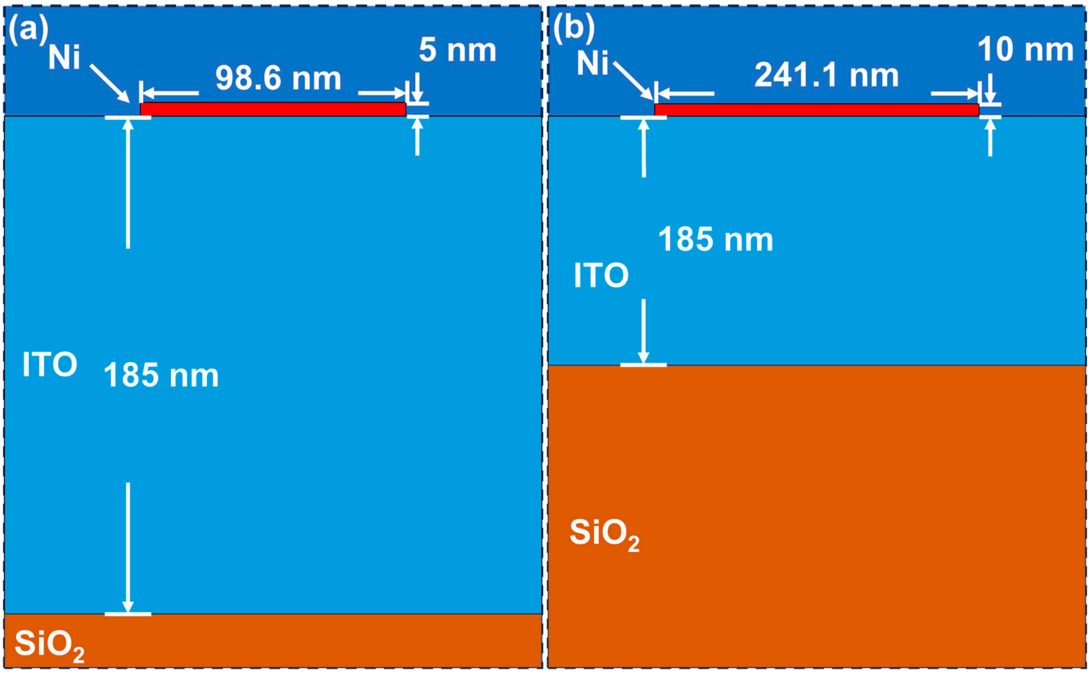

Figure S6. Schematic diagram of Ni nanofilms structure used in FDTD electric field simulation: (a)

5 nm Ni nanofilm, (b) 10 nm Ni nanofilm.

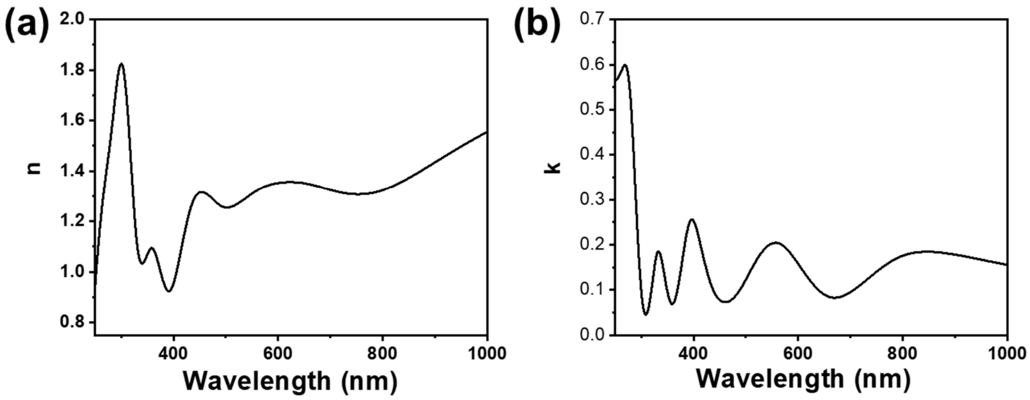

Figure S7. Measurement of optical constants of $NaYF_4$:Tm, Yb nanofilm: (a) n, (b) k.

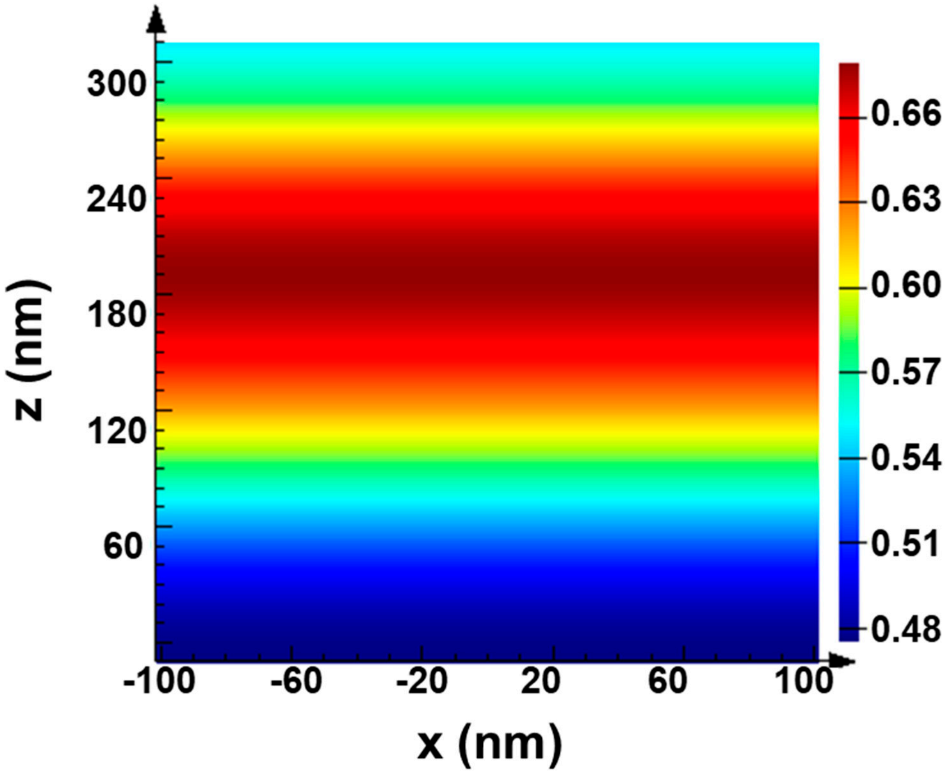

Figure S8. FDTD electric field simulation of the integrated Ni-UC nanofilm (5 nm Ni/ 350 nm $NaYF_4$: Tm, Yb).

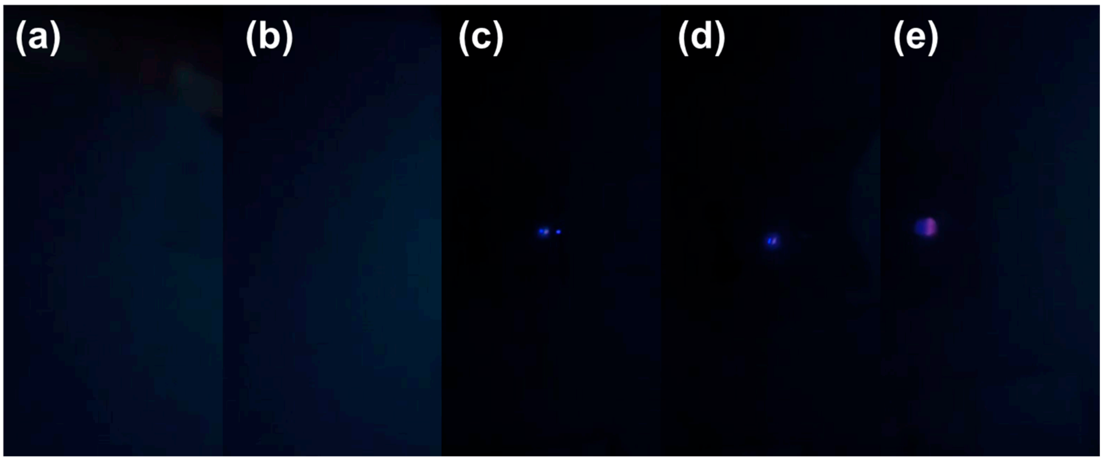

Figure S9. Photographs of fluorescence emission of different UC nanofilms and integrated Ni-UC nanofilms (we used a commercially available 100 mW laser pen emitting 980 nm laser to irradiate the UC nanofilms, filtered out the infrared light with a visible light filter, and photographed the UC nanofilms): (a) UC nanofilm (Unannealed), (b) UC nanofilm (400℃ annealing), (c) UC nanofilm (500℃ annealing), (d) Ni-UC nanofilm (5 nm Ni/ 350 nm $NaYF_4$:Tm, Yb), (e) Ni-UC nanofilm (10 nm Ni/ 350 nm $NaYF_4$:Tm, Yb).

Disclaimer/Publisher's Note: The statements, opinions and data contained in all publications are solely those of the individual author(s) and contributor(s) and not of MDPI and/or the editor(s). MDPI and/or the editor(s) disclaim responsibility for any injury to people or property resulting from any ideas, methods, instructions or products referred to in the content.
